# Hyper-Oxygenation Attenuates the Rapid Vasodilatory Response to Muscle Contraction and Compression

**DOI:** 10.3389/fphys.2018.01078

**Published:** 2018-08-15

**Authors:** Alessandro Messere, Michael Tschakovsky, Stefano Seddone, Gabriella Lulli, Walter Franco, Daniela Maffiodo, Carlo Ferraresi, Silvestro Roatta

**Affiliations:** ^1^Department of Neuroscience, University of Turin, Turin, Italy; ^2^Human Vascular Control Lab, School of Kinesiology and Health Studies, Queen’s University, Kingston, ON, Canada; ^3^Department of Mechanical and Aerospace Engineering, Politecnico di Torino, Turin, Italy

**Keywords:** muscle blood flow, hyperemia, muscle compression, muscle contraction, tissue oxygenation

## Abstract

A single muscle compression (MC) with accompanying hyperemia and hyper-oxygenation results in attenuation of a subsequent MC hyperemia, as long as the subsequent MC takes place when muscle oxygenation is still elevated. Whether this is due to the hyper-oxygenation, or compression-induced de-activation of mechano-sensitive structures is unclear. We hypothesized that increased oxygenation and not de-activation of mechano-sensitive structures was responsible for this attenuation and that both compression and contraction-induced hyperemia attenuate the hyperemic response to a subsequent muscle contraction, and vice-versa. *Protocol-1)* In eight subjects two MCs separated by a 25 s interval were delivered to the forearm without or with partial occlusion of the axillary artery, aimed at preventing hyperemia and increased oxygenation in response to the first MC. Tissue oxygenation [oxygenated (hemoglobin + myoglobin)/total (hemoglobin + myoglobin)] from forearm muscles and brachial artery blood flow were continuously monitored by means of spatially-resolved near-infrared spectroscopy (NIRS) and Doppler ultrasound, respectively. With unrestrained blood flow, the hyperemic response to the second MC was attenuated, compared to the first (5.7 ± 3.3 vs. 14.8 ± 3.9 ml, *P* < 0.05). This attenuation was abolished with partial occlusion of the auxillary artery (14.4 ± 3.9 ml). *Protocol-2)* In 10 healthy subjects, hemodynamic changes were assessed in response to MC and electrically stimulated contraction (ESC, 0.5 s duration, 20 Hz) of calf muscles, as single stimuli or delivered in sequences of two separated by a 25 s interval. When MC or ESC were delivered 25 s following MC or ESC the response to the second stimulus was always attenuated (range: 60–90%). These findings support a role for excess tissue oxygenation in the attenuation of mechanically-stimulated rapid dilation and rule out inactivation of mechano-sensitive structures. Furthermore, both MC and ESC rapid vasodilatation are attenuated by prior transient hyperemia, regardless of whether the hyperemia is due to MC or ESC. Previously, mechanisms responsible for this dilation have not been considered to be oxygen sensitive. This study identifies muscle oxygenation state as relevant blunting factor, and reveals the need to investigate how these feedforward mechanisms might actually be affected by oxygenation.

## Introduction

Since the pioneering work of Mohrman and Sparks (1974) the rapid hyperemia produced by a short lasting muscle compression (MC) has been shown to mimic the hyperemic response to a short-lasting contraction, the latter being often adopted as a model to investigate the hyperemia at the beginning of exercise (Valic et al., 2005). This similarity, originally observed in isolated muscles (Mohrman and Sparks, 1974) was later observed in several other studies in both animal (Turturici et al., 2012) and human studies (Kirby et al., 2007; Messere et al., 2017b). These observations suggested that the two hyperemia share common underlying mechanisms and that rapid dilatation at the beginning of exercise depends at least in part on the mechanical deformation of blood vessels (Clifford, 2007; Crecelius et al., 2013b; Jasperse et al., 2015).

The hyperemic response to MC was previously shown to exhibit a progressive attenuation in response to repetitive stimulation. This was first observed in the masseter muscle of the anesthetized rabbit (Turturici and Roatta, 2013a,b) and was later confirmed in the human forearm (Messere et al., 2017b). The effect was initially attributed to the possible inactivation of the putative mechano-sensitive vascular structures (Turturici and Roatta, 2013b; Messere et al., 2017b) according to similar inactivation patterns described for vascular mechano-sensitive structures (Honore et al., 2006; Earley and Brayden, 2015). However, the issue has been recently re-investigated in the calf muscles by means of an integrated experimental set-up that included simultaneous and continuous monitoring of arterial and venous blood flow, as well as local tissue oxygenation and blood volume by near infrared spectroscopy, addressing the effects of repetitive compressive stimulation at different frequencies [inter-stimulus interval (ISI): ranging from 20 to 160 s] (Messere et al., 2017a). The results demonstrated a dramatic reduction of the hyperemic response at ISI = 20 s, and a variable pattern of attenuation at larger ISIs (Messere et al., 2017a). Notably, the results showed that the attenuation was not dependent on the extent of filling of venous compartments while it was highly inversely correlated with the current oxygenation level in the muscles. This observation suggested that increased tissue oxygenation, as produced by the hyperemic response to the previous compressive stimulus/stimuli, may limit further hyperemia in the relevant tissue, thus supporting a primary role of tissue oxygenation in the control of muscle blood flow, even at the beginning of exercise (Golub and Pittman, 2013; Messere et al., 2017a).

However, the evidence of oxygenation-dependent attenuation of the compression-induced hyperemia was indirect since tissue oxygenation could not be altered independently of compressive stimulation of blood vessels and the possible concomitant inactivation of mechano-sensitive vascular structures could not be completely excluded.

A first aim of the present study was thus to isolate local tissue oxygenation changes from mechanical compression in the attenuation of compression-induced hyperemia. We hypothesized that if the compression-induced hyperemia were prevented by graded occlusion of the supplying artery this would prevent the increase in local tissue oxygenation as well as the attenuation of the hyperemic response to a subsequent compressive stimulus. This would rule out a deactivation of mechano-sensitive structures and support a role for tissue hyper-oxygenation in attenuating rapid vasodilatation.

In addition, we considered that: (1) the contraction-induced hyperemia is believed to share the same mechanisms as the compression-induced hyperemia and (2) the short lasting contraction has also been shown to produce a transient increase in tissue oxygenation (Towse et al., 2011), just like the MC (Messere et al., 2017a). A second aim of this study was then to test whether the increase in tissue oxygenation also affects the contraction-induced hyperemia. We hypothesized that, irrespective on whether the local increase in tissue oxygenation was produced by a prior contraction or compression it would have attenuated the response to a subsequent contraction or compression.

## Materials and Methods

### Subjects

Eight healthy men (age: 32.6 ± 9.3 years; weight: 74.1 ± 9.2 kg; height: 180.5 ± 9.9 cm) were enrolled in *protocol 1*, and 10 healthy subjects (9 men and 1 women; age: 26.7 ± 3.5 years; weight: 68.2 ± 7.6 kg; height: 174.6 ± 6.1 cm) in *protocol 2*. All subjects were non-obese and normotensive (resting blood pressure <140/90 mmHg).

This study was carried out in accordance with the International Ethical Guidelines for Biomedical Research Involving Human Subjects, prepared by the Council for International Organizations of Medical Sciences. The protocol was approved by the Ethical Committee of the University of Turin. All subjects gave written informed consent in accordance with the Declaration of Helsinki.

### Experimental Setup

#### Near-Infrared Spectroscopy

A continuous wave near-infrared spectroscopy (NIRS) device (NIRO-200NX, Hamamatsu Photonics, Hamamatsu City, Japan) was used to measure local hemodynamic changes. The device implements both the classical modified Lambert–Beer method (Ferrari et al., 2011) and the spatially-resolved spectroscopy methods (Matcher et al., 1995). Based on our previous experience we focused our attention on spatially-resolved parameters which, being less affected by cutaneous circulation, provide a more specific monitoring of muscle tissue hemodynamics (Canova et al., 2011; Messere and Roatta, 2013, 2015). The device provides an indicator of tissue oxygenation [tissue oxygenation index (TOI)], representing the percentage ratio of oxygenated to total hemoglobin. However, since NIRS cannot discriminate between hemoglobin and myoglobin, all measurements actually refer to the whole (hemoglobin + myoglobin) concentration (Spires et al., 2011). The NIRS probe (inter-optodes distance: 4 cm) was located over wrist extensor muscles (flexor carpi radialis and ulnaris) of the left forearm, in *protocol 1*, and over the belly of the lateral head of the gastrocnemius muscle of the right leg in *protocol 2*.

#### Echo-Doppler Sonography

Measurements of vessel diameter and blood velocity were performed at the brachial artery of the left arm at about mid-way between the shoulder and the elbow in *protocol 1* and at the superficial femoral artery of the right leg distally to the inguinal ligament for *protocol 2* by means of an Echo-Doppler device (Mylab 25, Esaote, Genoa, Italy), equipped with a 12 MHz linear array (LA 523, Esaote, Genoa, Italy). Artery diameter was determined, as the average of three measurements, taken perpendicularly along the central axis of the insonated area, at the beginning of every protocol. Blood velocity was measured continuously throughout the protocols, with an insonation angle of about 60° (operating frequency of 5 MHz) and with the sample volume extended to include the entire vessel diameter.

#### Electrical Muscle Stimulation

In *protocol 2* only, two self-adhesive surface electrodes (4.5 cm × 8.0 cm) were placed transversely at the proximal and distal ends of the lateral gastrocnemius muscle of the right leg. An electrical stimulator (DS7A, Digitimer, Letchworth Garden City, United Kingdom) was used to deliver trains of square-wave pulses (pulse duration: 500 μs; frequency: 20 Hz; total train duration: 0.5 s; intensity of stimulation: supramaximal).

Supramaximal intensity of stimulation was determined as the current intensity at which the maximum contraction force was achieved, further increased by 15%. The muscle was maintained in isometric conditions, the knee flexed at 90° and blocked by means of straps attached to the ground. The force level of the evoked contraction was measured by a force transducer (TF 031, CCt Transducers, Turin, Italy) inserted in-series with the straps.

The straps were removed at the end of this procedure, so that some movement of the joint was allowed during electrical stimulation. Duration and frequency of the electrical stimulation were established based on preliminary experiments aimed at obtaining a similar hyperemic response to that of MC.

#### Limb Compressions

In *protocol* 1 compression of the forearm (suprasystolic 250 mmHg, duration: 1 s) was delivered by a blood pressure cuff (Gima, Gessate, Italy) wrapped around the forearm and connected to an air compressor by means of electrical solenoid valves controlled by the computer (Messere et al., 2017b).

In *protocol 2* compression of the leg was achieved by a custom-made cuff that was developed for an intermittent pneumatic compression system, consisting of four different bladders (Ferraresi et al., 2014, 2016; Messere et al., 2017a) All bladders were inflated simultaneously to a supra-systolic pressure of 150 mmHg (in about 2–3 s), and rapidly deflated. Squared-wave output analog signals were generated by the system to indicate inflation and deflation times.

**FIGURE 1:**
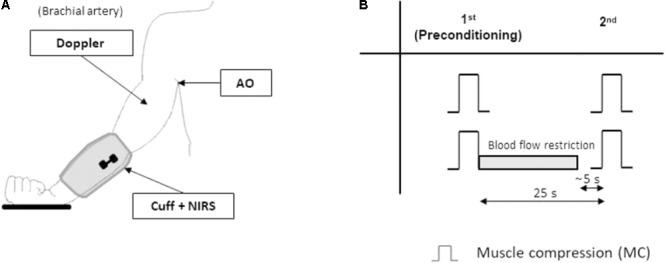
*Protocol 1.* Experimental setup and protocol. The experimental setup **(A)** for *protocol 1* includes: a blood pressure cuff (Cuff) to deliver repeatable muscle compressive (MC) stimuli to the forearm, the near-infrared spectroscopy (NIRS) probe over the wrist extensor muscles, and the echo-Doppler probe, monitoring blood flow in the brachial artery. More proximally, the axillary artery can be partially occluded by the operator (artery occlusion AO) to restrict blood flow. The experimental protocol **(B)** includes two randomized sequences of two muscle compressions (MC) separated by a 25 s interval. In one of the sequences the hyperemic response to the first (pre-conditioning) MC is prevented by partial occlusion of the axillary artery (gray bar). Note that partial occlusion of the artery is interrupted about 5 s before the second MC.

**FIGURE 2:**
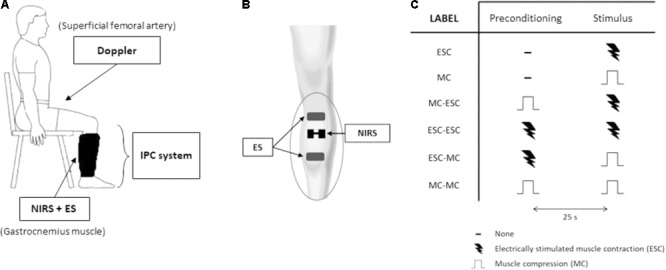
*Protocol 2.* Experimental setup and protocol. The experimental setup **(A)** includes an intermittent pneumatic compression system for the compression of the lower limb, echo-Doppler monitoring of blood flow at the femoral artery, near-infrared spectroscopy (NIRS) monitoring over the lateral head of gastrocnemius muscle and electrical stimulation (ES) of the same muscle. Arrangement of NIRS probe and ES electrodes on gastrocnemius muscle, underneath the pneumatic cuff **(B)**. The experimental protocol **(C)** consists of a randomized series of sequences of two stimuli separated by a 25 s interval: muscle compression (MC) at 150 mmHg and an electrically-stimulated contraction (ESC) (0.5 s duration, at 20 Hz). The table depicts all tested sequences.

Both the NIRS probe and the stimulating electrodes were positioned on the limb under the pneumatic cuff as previously described (Messere et al., 2017a,b) without disturbing the compression nor providing discomfort (**Figures 1A, 2A,B**).

### Experimental Protocols

The experimental protocols were performed in a quiet room with a constant ambient temperature of about 21–23°C. The subject sat upright on an adjustable chair with the back supported by a back rest.

#### Protocol 1

This experimental protocol was aimed to test the hypothesis that the increased tissue oxygenation produced by the hyperemia to a first compressive stimulus is responsible for the attenuation of the hyperemia produced by a second compressive stimulus delivered 25 s later. An inter-stimulus interval of 25 s was chosen because it was shown to be long enough for blood flow to return to resting level and tissue oxygenation was observed to peak at about that time in both calf (Messere et al., 2017a) and forearm muscles in preliminary experiments and to slowly decline afterward.

The left arm of the subject was relaxed down, slightly abducted (approximately 15–20°), the elbow about 10° flexed, with the hand laying on a pillow, the palm upward. A cooling glove (TM7001 Therapy Mitten, Elasto-Gel, North Kansas City, MO, United States) was used to minimize the contribution of skin blood flow. The relaxed forearm was subjected to a sequence of two compressive stimuli separated by a 25 s interval. This sequence was repeated twice: in one occasion no limitation to the hyperemia produced by the first compressive stimulus was implemented; in the other, the hyperemia was prevented by concomitant partial occlusion of the brachial artery (Pyke et al., 2008) at the level of the insertion of the biceps brachii muscle on the humerus (**Figure 1B**) as follows. At the beginning of the series subject’s axillary pulse was located by the thumb of the examiner, just distal to the axillary fossa. Immediately after the first compressive stimulus, by adjusting the pressure exerted by the thumb, the examiner could prevent the increase in blood flow in the brachial artery. Continuous monitoring of blood velocity in the brachial artery during the maneuver was used by the examiner as a feedback to adjust the level of the applied pressure. The pressure over the artery was completely released about 5 s before the occurrence of the second forearm compression, in order to prevent interference with the subsequent hyperemia.

#### Protocol 2

This experimental protocol was aimed at testing the hypothesis that increased tissue oxygenation either produced by prior compression or contraction of the relevant muscles affects the rapid vasodilatation-mediated hyperemic response to a subsequent compression or contraction of the same muscles.

After 15 min of rest the hemodynamic changes were assessed in response to: (1) a single (unconditioned) electrically-stimulated contraction (ESC); (2) a single (unconditioned) MC; (3) a MC followed by an ESC, 25 s later; (4) an ESC followed by an ESC; (5) an ESC followed by a MC; and (6) a MC followed by a MC (**Figure 2C**). These stimulations were delivered in random order and separated by resting intervals of at least 4 min.

### Data Acquisition and Processing

The NIRS analog output was digitally acquired (CED Micro 1041, Cambridge Electronic Design, Cambridge, United Kingdom) along with Doppler audio signals and the synchronism signal from the intermittent pneumatic compression device and stored on the computer for later analysis with Spike2 software (version 6.10, Cambridge Electronic Design, Cambridge, United Kingdom).

A specific algorithm was implemented in the Spike2 script language to calculate blood velocity from the Doppler audio signal (Messere et al., 2017a,b). Briefly, the weighted mean frequency of the power spectrum of the Doppler shift was assessed, averaged over each cardiac cycle and converted to blood velocity (Vmean, expressed in cm/s). Blood flow (BF, in ml/min) was calculated as BF = Vmean ^∗^ π ^∗^ (vessel diameter/2)^2 ∗^ 60.

The responses induced by both MC and ESC were assessed in terms of changes in arterial blood flow and local tissue oxygenation. Three parameters were calculated for both variables: (1) the baseline (pre-stimulus) value (BF-bsl and TOI-bsl), calculated as the time-average over the 20 s interval preceding the unconditioned stimulus (or the 3 s interval preceding the pre-conditioned stimulus); (2) the peak value (BF-peak and TOI-peak); and (3) the time to peak, which refers to time from the end of the mechanical or electrical stimulation to the peak response (BF-tpeak and TOI-tpeak).

The duration of the hyperemic response (Hyp-duration) was identified as the time interval in which blood flow remained above baseline by at least 10% of the difference between the baseline and the peak. In addition, the magnitude of the hyperemia was quantified as the excess blood volume (EBV) received by the tissues during the hyperemia: EBV = time integral of (BF – BF-bsl) computed over the duration of the hyperemia.

The changes observed in tissue oxygenation were expressed as ΔTOI = TOI-peak – TOI-bsl. In addition the speed of the decay in tissue oxygenation after the end of the hyperemia was quantified as the slope of TOI tracing (TOI-slope) measured over a 20 s time interval starting 25 s after the end of the unconditioned or pre-conditioned stimulus.

### Statistical Analysis

Changes in blood flow and tisue oxygenation induced by a single stimulation (i.e., MC in *protocol 1*, and ESC and MC in *protocol 2*) were assessed by a *t*-test. In addition paired *t*-tests were performed for *protocol 2* to evaluate differences between unconditioned MC and ESC in changes induced in blood flow and tissue oxygenation.

To examine the effect of pre-conditioning, a one-way ANOVA for repeated measures was performed with type of pre-conditiong (with or without blood flow restriction) as factor for *protocol 1* and a two-way ANOVA for repeated measures was performed for *protocol 2*, with factors the pre-conditioning stimulus (none, MC, or ESC) and the conditioned stimulus (MC or ESC). When significance was found, a Duncan’s *post ho*c test was performed to assess significant differences. All statistical analyses were performed using commercially available software (GraphPad Prism v 6.0, GraphPad Software, La Jolla, CA, United States). The level of statistical significance was set at *P* < 0.05. All data are expressed as means ± standard deviation in the text and means ± standard error in the table and diagrams.

## Results

### Protocol 1: Pre-conditioning Effects of Hyperemia

**FIGURE 3:**
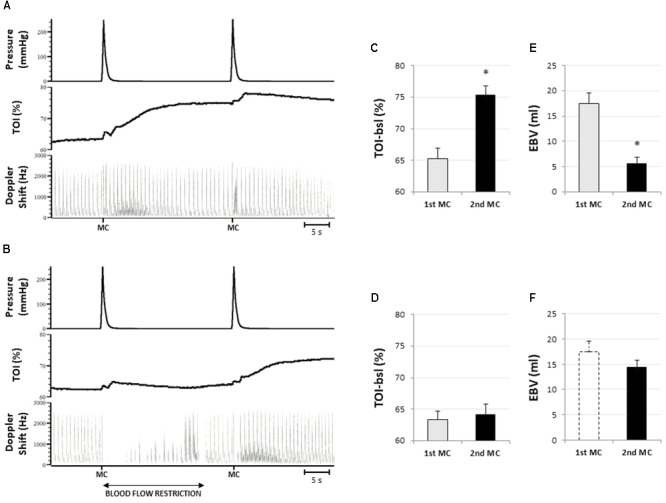
Representative responses and results of *protocol 1.* **(A)** Original tracings from a representative subject showing the response to two subsequent compressive stimuli (MC) from top to bottom: cuff pressure, tissue oxygenation in forearm muscles (TOI), and blood flow in the brachial artery (represented by the Doppler shift). Note that the hyperemic response to the second stimulus is strongly attenuated. In **(B)** the hyperemia produced by the first compressive stimulus is prevented by restriction of the blood supply. Note that the hyperemic response to the second stimulus is fully expressed. The bar diagrams indicate the pre-stimulus tissue oxygenation level **(C,D)** and the amplitude of the hyperemic response **(E,F)** to the first (1st MC) and the second stimulus (2nd MC), without **(C,E)** or with **(D,F)** blood flow restriction. Amplitude of the hyperemia is expressed as excess of blood volume (EBV). The dashed bar in **(F)** replicates the EBV response to the 1st MC without blood flow restriction (as presented in **E**), for comparison. ^∗^Significantly different from 1st MC (*P* < 0.05; *n* = 8).

As shown by the original tracings of **Figure 3A**, the first MC at the forearm produces the typical compression-induced hyperemia, which is followed by a slower and longer-lasting increase in tissue oxygenation. Brachial artey blood flow (49.7 ± 9.4 ml/min) increased after MC, peaking at 136.5 ± 45.3 ml/min (*P* < 0.01). The total hyperemic response lasted 10.3 ± 3.9 s, and resulted in EBV of 17.4 ± 6.0 ml. Tissue oxygenation slowly increased from baseline (65.2 ± 4.7%), to a maximum of 76.5 ± 3.8% (*P* < 0.01) after MC.

A second MC delivered 25 s later takes place when blood flow is returned to baseline level while TOI is still elevated (pre-compression TOI: 75.3 ± 4.2%) (**Figures 3A,C**). The hyperemic response in this condition was shortened in duration, to 5.3 ± 2.5 s (*P* < 0.05) and largely attenuated: on average peak blood flow decreased to 75.1 ± 30.8 ml/min (*P* < 0.05) and EBV decreased to 5.7 ± 3.3 ml (*P* < 0.05) (**Figure 3E**).

When the hyperemic response to the conditioning stimulus was prevented by partial occlusion of the supplying artery the TOI remained unchanged until the second stimulation (pre-compression TOI: 64.9 ± 4.9 vs. 63.3 ± 3.9%) (**Figures 3B,D**). In this condition the hyperemia was not significantly different from the one produced by the first MC in terms of peak (120.9 ± 32.8 ml), duration (9.7 ± 2.2 s), and EBV (14.4 ± 3.9 ml) (**Figure 3F**).

Note that the artery occlusion is released about 5 s before the delivery of the second stimulus and that blood flow is returned at about resting level by that time (**Figure 3B**), thus excluding the possible occurrence of a reactive hyperemia.

The bar diagrams in **Figure 3** illustrate the inverse relationship between tissue oxygenation level at the time of MC (TOI-bsl) and the extent of the hyperemic response (EBV).

### Protocol 2: Comparison of Compression- and Contraction-Induced Hyperemia

**FIGURE 4:**
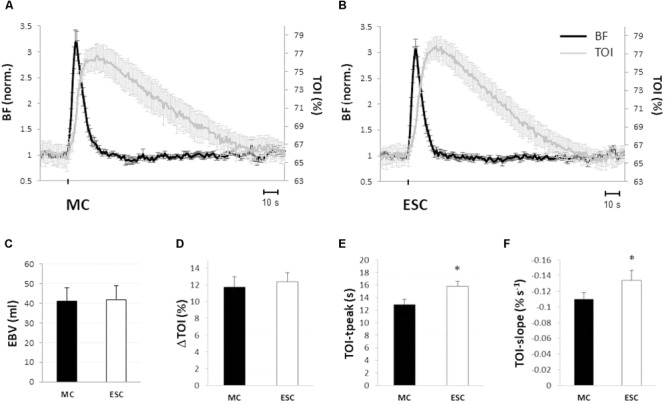
Hemodynamic responses to a single muscle compression (MC) and electrical stimulated contraction (ESC). Average hemodynamic response to a single MC **(A)** and an ESC **(B)** (*protocol 2*): femoral artery blood flow (BF, black line), and tissue oxygenation at gastrocnemius muscle (TOI, gray line). Changes in BF are normalized to baseline. Note the similarity of the response of both variables to MC and ESC. Hemodynamic responses to MC (black) and ESC (white) are compared in terms of: magnitude of the hyperemic response (expressed as EBV, excess of blood volume) **(C)**, increase of tissue oxygenation (peak – baseline) **(D)**, the absolute peak in tissue oxygenation **(E)** and its rate of decrease (slope) during return toward baseline **(F)** (*n* = 10). ^∗^Significantly different from MC (*p* < 0.05).

By design, hemodynamic responses to single MC and ESC were strikingly similar in both amplitude and time course of BF and TOI, as can be observed from the average tracings of **Figure 4**. Baseline blood flow assessed at the femoral artery was similar before both stimuli (135.9 ± 34.0 and 131.4 ± 32.3 ml/min, for the MC and the ESC, respectively) and increased up to 428.4 ± 112.4 and 415.0 ± 103.1 ml/min peaking at 3.3 ± 0.8 and 3.0 ± 0.4 s after the stimulation, respectively. The hyperemic response lasted 16.8 ± 4.8 and 16.3 ± 2.4 s, respectively, for MC and ESC, and was quantified by an EBV of 41.0 ± 15.5 and 41.9 ± 12.4 ml, respectively (**Figure 4C**). In none of the above measurements was the response to ESC significantly different from MC.

Both stimuli produced a comparable transient hyper-oxygenation in the tissue, increasing TOI by 11.8 ± 4.2 and 12.8 ± 3.7% (**Figure 4D**) for MC and ESC, respectively. The only significant difference between the responses to MC and ESC was observed in the time course of TOI, peaking after 12.7 ± 2.9 s from deflation and after 15.9 ± 2.2 s from the end of ESC (*P* < 0.05) (**Figure 4E**). In addition the TOI return to basal level was slower for MC (slope of -0.11 ± 0.03%/s) than for ESC (-0.13 ± 0.04%/s) (**Figure 4F**).

### Protocol 2: Effects of Pre-conditioning Stimulation

**FIGURE 5:**
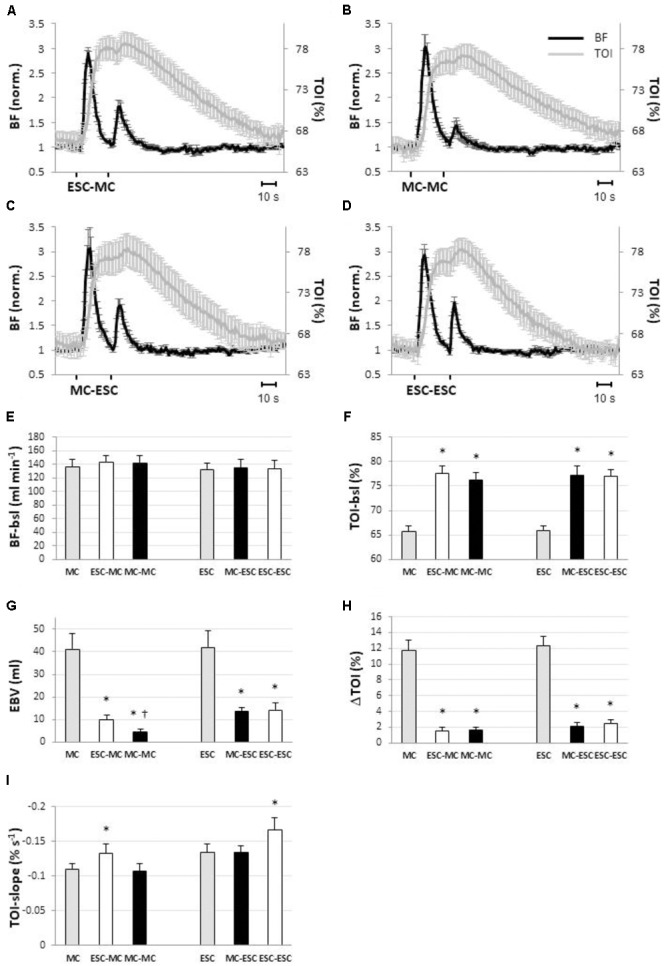
Hemodynamic responses observed in blood flow (BF, black line) and in tissue oxygenation index (TOI, gray line) in response to different sequences of muscle compressions (MC) and electrical stimulated contractions (ESC) (*protocol 2*): ESC-MC **(A)**, MC-MC **(B)**; MC-ESC **(C)**; ESC-ESC **(D)**. Within each sequence stimuli are separated by a 25-s interval (*n* = 10). The bar diagrams show how preconditioning by ESC (white) and MC (black) affected pre-stimulus level of blood flow **(E)** and tissue oxygenation **(F)**, excess blood volume (EBV) **(G)**, increase in tissue oxygenation (TOI) **(H)** and slope of the return curve of tissue oxygenation **(I)** in the different stimulation sequences. Basal levels (i.e., pre-stimulus levels) and the responses to the single (without preconditioning) MC and ESC stimuli are also given for reference (gray). Note that blood flow is unaffected by preconditioning while TOI is elevated irrespective of the preconditioning stimulus. In addition TOI return slope **(C)** is increased in proportion to the number of ESCs in the sequence. ^∗^Significantly different from single stimulation (*P* < 0.05). ^†^Significantly different from all other conditions (*P* < 0.05); (*n* = 10).

The responses to conditioned stimuli are shown in **Figure 5**. It is apparent that pre-conditioning by prior delivery of either MC or ESC strongly attenuates the response to both MC and ESC. **Figure 5** also shows basal values for BF (**Figure 5E**) and TOI (**Figure 5F**) for the four pre-conditioned stimuli, in comparison to the single (unconditioned). It can be observed that was completely returned to baseline before the delivery of the pre-conditioned stimulus, i.e., it was not significantly affected by the pre-conditioning (**Figure 5E**) while tissue oxygenation remained increased in each condition (*P* < 0.05) (**Figure 5F**).

In **Figure 5G** the extent of attenuation of the hyperemic response in the different conditions can be observed: EBV in MC-induced hyperemia was reduced to 9.8 ± 2.1 ml (*P* < 0.05) when pre-conditioned by ESC (i.e., ESC-MC) and to 4.5 ± 1.2 ml (*P* < 0.05) when pre-conditioned by MC (i.e., MC-MC). Also the ESC-induced hyperemia was reduced when conditioned by MC (i.e., MC-ESC, 13.6 ± 3.6 ml, *P* < 0.05) and ESC (i.e., ESC-ESC, 14.2 ± 3.4 ml, *P* < 0.05).

Along the same line, the increase in tissue oxygenation was reduced by conditioning, with respect to the single stimulation (**Figure 5H**). In particular, for MC the increase was reduced to 1.6 ± 1.4% for ESC-MC (*P* < 0.05) and to 1.6 ± 1.2% (*P* < 0.05) for MC-MC. Similarly, a reduction of increased tissue oxygenation was also observed when ESC was conditioned [2.1 ± 1.6% (*P* < 0.05) and 2.4 ± 1.7% (*P* < 0.05) for MC-ESC and ESC-ESC, respectively].

As for the time course of TOI, the TOI-slope during recovery was significantly increased by ESC pre-conditioning with respect to the unconditioned (**Figure 5I**) stimulation (ESC-MC: -0.13 ± 0.03%/s, *P* < 0.05; and ESC-ESC: -0.17 ± 0.05%/s, *P* < 0.05). Otherwise no changes were observed, in fact the slope values were -0.11 ± 0.04%/s and, -0.13 ± 0.03%/s, respectively, for MC-MC and MC-ESC.

## Discussion

The present study disclosed several new features of the rapid hyperemia produced by a short lasting MC as well as by a muscle contraction. In particular it showed that: (1) muscle contraction and compression may produce a very similar hemodynamic response, in terms of both the rapid hyperemia and the ensuing slower rise in tissue oxygenation; (2) a single short-lasting compression or contraction may strongly attenuate the subsequent hyperemic response to both a compression or a contraction of the same muscles, performed 25 s later; (3) this effect is almost abolished if the hyperemic response to the first stimulus and the ensuing increase in tissue oxygenation are prevented by a partial occlusion of the supplying artery. The results demonstrate for the first time that the increase in tissue oxygenation and not the possible inactivation of mechano-sensitive vascular structures is responsible for the attenuation of the hyperemic response to subsequent stimuli. A novel role is disclosed for tissue oxygenation as major modulator of the vasodilatory mechanisms underlying the rapid hyperemic response to both muscle contraction and compression.

### Role of Tissue Oxygenation in the Rapid Dilatation Produced by Mechanical Stimulation

It is generally accepted that a rapid dilatation can be elicited by mechanical stimulation of the vascular network and contribute to the rapid hyperemia that is observed in response to passive movement (Turturici et al., 2012; Broxterman et al., 2017) or pneumatic compression (Brock et al., 1998; Kirby et al., 2007; Turturici et al., 2012) of the muscle as well as to active muscle contraction (Kirby et al., 2007; Clifford and Tschakovsky, 2008; Turturici et al., 2012; Crecelius et al., 2013a). However, the actual mechano-sensitive structures and vascular sites have not yet been identified. A number of studies have implicated nitric oxide, potassium ions, and prostaglandins (Armstrong et al., 2007; Casey et al., 2013; Crecelius et al., 2013a) but the possible underlying signaling pathways are still unknown. To this constellation of mechanisms, the present study demonstrates for the first time that tissue oxygenation can profoundly modulate the rapid dilatation.

A first observation of this phenomenon was recently reported (Messere et al., 2017a). When delivering a sequence of compressive stimuli to the calf muscles the hyperemic response to each single stimulus was shown to correlate with the level of oxygenation present in the tissue at the time of stimulus delivery (Messere et al., 2017a). The results of *protocol 1* show that the hyperemic response to a second MC delivered 25 s after a first one is attenuated by 70% and that this attenuation disappears if the hyperemia and the ensuing increase in oxygenation are prevented by a partial occlusion of the supplying artery. Since the mechanical stimulation of the vasculature is unchanged in the two conditions, these results rule out mechano-sensitive vascular structure inactivation by prior MC and that the occurrence of a prior hyperemia and hyper oxygenation is necessary for the attenuation of the hyperemic response to the second stimulus.

This observation fits with the “bang-bang” model recently advanced by Golub and Pittman (2014). According to this model, tissue oxygenation would be the pivotal variable for the control of muscle blood flow. In short, the authors hypothesized that increased tissue pO_2_ would raise the concentration of superoxide anion O_2_- that would in turn inactivate NO and its dilatory action, thus reducing blood flow and restoring the basal tissue pO_2_ level (Golub and Pittman, 2014). In several studies pharmacological blockade of NO synthase by NG-monomethyl-L-arginine has been shown to attenuate 20–90% the rapid hyperemic responses to different stimuli such as, contraction (Brock et al., 1998; Casey et al., 2013), compression (Brock et al., 1998) and passive movement (Mortensen et al., 2012; Trinity et al., 2012; Broxterman et al., 2017), which is comparable with the extent of attenuation of the hyperemic responses to MC and contraction here observed, ranging from 60 to 90% in the different conditions. On this basis, the above hypothesis offers a plausible pathway for the modulatory role of tissue oxygenation on the rapid dilatation. However, in some of the studies the attenuation of the hyperemic response was in fact rather small while others failed to evidence a significant attenuation of the rapid response to passive movement and exercise by NO synthase blockade (Radegran and Saltin, 1999). Thus, the actual involvement of this pathway in the attenuation of the rapid dilatation by hyperoxia remain to be ascertained.

Support to this role for hyperoxia also comes from the early study by Fairchild et al. (1966) showing that reactive hyperemia remains elevated if the ischemic limb is re-perfused with anoxic blood, indicating that recovery of tissue oxygenation is necessary for the recovery of vascular tone (Fairchild et al., 1966). The present results integrate this picture showing that hyper-oxygenation may effectively prevent further hyperemic events.

The lack of this local regulatory mechanism in isolated vessel preparations likely explains why, when multiple compressive stimuli are delivered to isolated feed arteries, a potentiation of the dilatation is observed (Clifford et al., 2006) rather than an attenuation, as reported in the present and other studies in humans and intact animals (Tschakovsky et al., 1996; Turturici and Roatta, 2013a; Messere et al., 2017a,b).

Although tissue oxygenation is generally considered to be a relevant factor in the control of muscle blood flow it may be surprising to observe such a prompt and prominent counter-regulatory action by hyperoxia on hyperemic responses, as reported in the present study. In fact, increasing arterial pO_2_ produces in general modest flow-limiting effects on resting blood flow and functional hyperemia (Welch et al., 1977; Casey et al., 2011). However, it must be observed that arterial hyperoxia may not be very effective in increasing tissue pO_2_, given that hemoglobin is already almost saturated at normal pO_2_ levels. In contrast, with the muscle directly exposed to elevated pO_2_ (150 mmHg) a reduction of resting capillary blood velocity by 75% and of the response to a120 s artery occlusion by 44% was observed (Tuma et al., 1977). Notably, during a compression-induced hyperemia the amount of oxygen transported to the tissue is increased in proportion to blood flow. The peak flow in compression-induced hyperemia has been reported to be 200–360% of baseline when assessed in large arteries in humans (Kirby et al., 2007; Credeur et al., 2015; Messere et al., 2017a,b) and up to 500% of baseline, when assessed in a purely muscular artery, in the rabbit (Turturici et al., 2012) thus implying an equivalent increase in oxygen delivery to the tissue. Such abnormally-increased O_2_ supply may lead to the relevant increase in tissue pO_2_, that was detected by TOI in the present and the previous study (Messere et al., 2017a).

### Pre-conditioning of Contraction-Induced Hyperemia and Functional Implications

A novel finding in the present study is that the hyperemic response to contraction is drastically attenuated by a short compression delivered to the relevant muscles 25 s earlier. In light of what was previously discussed, this effect stems from the increase in tissue oxygenation generated by the compressive stimulus. On the one hand this confirms the preliminary observation that the hyperemic response to electrically-stimulated contraction of forearm muscles is attenuated by a repetitive pneumatic compression of the forearm (Messere et al., 2017b), on the other it discloses a novel feature of tissue oxygenation, i.e., the capacity of effectively preventing rapid (mechanically-induced) hyperemia. In fact, tissue oxygenation is well-known to have a role in matching blood-flow to metabolism, in particular by operating a prompt dilatation in response to a local decrease in tissue oxygenation. On the contrary, counteracting hyper-perfusion is generally not considered a priority or a particularly prominent feature of local metabolic mechanisms, at least in skeletal muscle. This may partly be due to the difficulty in discriminating myogenic from metabolic mechanisms in response to step changes in perfusion pressure, as investigated in classical studies (Folkow, 1949; Jones and Berne, 1964). The present results contradict this belief, and show that a condition of local hyper-oxygenation (obtained in the absence of transmural pressure changes) does exert a powerful action against further hyper-perfusion, as produced by the rapid dilatation.

Generally, the rapid onset vasodilation at the beginning of exercise is considered to act as a feed-forward mechanism, anticipating the increase of metabolic needs (Clifford, 2007; Kirby et al., 2007). Prevention of feed-forward dilatation could serve both saving of system resources and maintaining tissue homeostasis, while protecting from the possible adverse effects of hyper-oxygenation.

### Contraction- vs. Compression-Induced Hyperemia

The hyperemic response to MC is generally reported to be lower than to contraction although both responses depend on the intensity and duration of the compression or contraction, respectively (Kirby et al., 2007; Credeur et al., 2015). In the present study by adjusting stimulation frequency and duration of supramaximal ESC we could easily elicit a hyperemic response of the same magnitude as MC. This allowed us to demonstrate that in this case the magnitude of response of TOI is also comparable in the two conditions. The increase in tissue oxygenation in response to ESC confirms previous observations based on NIRS and magnetic resonance (Towse et al., 2011) and supports the concept that the rapid onset hyperemia is not driven by the increased metabolism creating a mismatch between oxygen demand and delivery (which would be associated with a decrease in tissue oxygenation) (Gorczynski and Duling, 1978). Small significant differences where, however, detected: the TOI response to ESC peaks slightly later and returns to control slightly faster than with MC. This difference may be attributed to the oxygen debt that is contracted during the contraction and payed afterward, thus accelerating the return from hyper-oxygenation.

In this regard, it is important to note that the extent of attenuation of the hyperemic response is larger in the MC-MC sequence, than in all others sequences of *protocol 2*. It can be hypothesized that repayment of the oxygen debt contracted during ESC consisting of an increased oxygen flow from capillaries to muscle cells is accompanied (actually caused) by the development of the necessary pO_2_ gradients, resulting in a lowering of interstitial pO_2_. This could be responsible for a weaker attenuation produced in all the sequences including an ESC.

It must also be noted that the negative slope of TOI after the ESC-MC is the same as after the single ESC, in spite of the fact that it is assessed 25 s later, i.e., 55 s after the contraction. Moreover, as compared to MC (no contraction), the increase in slope after ESC-ESC (two contractions) is twice as large compared to after ESC, MC-ESC, and ESC-MC (one contraction) which confirms the observation that the effects of the first contraction are still present about 1 min later (**Figure 5I**). Given that blood flow is basically returned to stable control levels when TOI exhibits its linear decay, we can reasonably inteprt the slope of TOI decay as an indicator of tissue metabolism. Those observations imply that, in spite of the hyperemia that exceeds metabolic needs, the “oxygen debt” associated with ESC takes longer than 1 min for repayment. This indication appears to be compatible with rates of phosphocreatinine resynthesis: a time constant of 63 s was measured in forearm flexors muscle recovering from ischemia (Blei et al., 1993), and of 68 s in calf muscles after high intensity exercise (McCully et al., 1994), while a faster recovery was observed after light exercise or in trained individuals (McCully et al., 1994; Yoshida et al., 2013), e.g., half-time of phosphocreatinine concentration recovery of about 15 s was reported in finger flexors after a short period (18 s) of light exercise (Bendahan et al., 2003). All these studies analyzed phosphocreatinine resynthesis and/or tissue re-oxygenation, after a more or less marked decrease in phosphocreatinine concentration and tissue oxygenation. It is surprising that in the present study the same phenomenon could be detected in response to a very short contraction (0.5 s, 20 Hz) and in a condition of hyper-perfusion and hyper-oxygenation.

### Reciprocal Pre-conditioning Between Contraction- and Compression-Induced Hyperemia

The results of *protocol 2*, depicted in **Figure 5**, clearly describe a reciprocal interaction between muscle contraction and compression, i.e., that the attenuation of the hyperemic response to the second stimulus occurred in all possible sequences of two stimuli, irrespective of the actual sequence.

In light of what was previously discussed these results can be easily interpreted: both stimuli produce a similar hyperemia with the ensuing increase in tissue oxygenation that effectively attenuates the hyperemic response to the subsequent stimulation. On the same basis some previous findings concerning vascular responses to sequences of mechanical stimuli can now be reinterpreted. For instance both the short-lasting occlusion of the supplying artery (Turturici et al., 2012) and the stretch of the passive muscle (Turturici et al., 2012; Venturelli et al., 2017) were shown to elicit a rapid hyperemia that exhibited progressive attenuation upon repeated stimulation (Turturici and Roatta, 2013b; Venturelli et al., 2017). Since these hyperemia took place in the passive muscle they were most likely associated with increased tissue oxygenation. Thus we would propose that the oxygenation-driven restriction of blood flow applies more generally to any mechanically-induced hyperemia, however, evoked in the passive muscle. Obviously the attenuation of the hyperemia does not take place during repeated active contractions in which tissue oxygenation is known to decrease.

### Limitations

In principle, a hyperemia developing in the absence of an increase in metabolism, besides increasing tissue oxygenation, may alter other tissue variables, e.g., it may decrease CO_2_ concentration and increase pH. Although their precise role in the regulation of muscle blood flow is not known, a possible contribution to the observed effects cannot be excluded on the basis of the present data.

The TOI, while having the advantage to focus on tissues in depth, i.e., the muscles (Messere and Roatta, 2013, 2015), derives the measurement from the whole sample volume, i.e., including arterial and venous vessels. Thus, its measurement is not specifically related to the microcirculation and may be affected by blood oxygenation changes, particularly in venous compartments, which are likely to take place in this kind of experiment. New technologies and procedures will be needed to provide more specific assessment of tissue pO_2_.

A further limitation relates to the fact that the subject pool is not gender balanced thus the issue of possible gender-related as well as age-dependent differences needs to be addressed in future studies.

## Conclusion

The present study provides direct evidence of the novel role of tissue oxygenation as a modulator of the rapid dilatory mechanisms underlying the hyperemic responses to both muscle contraction and compression. This is responsible for the attenuation of the hyperemic response occurring during repetitive mechanical stimulation. For the first time it has been shown that the increase in tissue oxygenation can limit the contraction induced hyperemia, thus limiting the feed forward dilatation at the onset of the exercise.

In light of the present findings, it is suggested that current tissue oxygenation levels are taken into account for better understanding vascular reactivity to fast hemodynamic transients.

## Data Availability

The raw data supporting the conclusions of this manuscript will be made available by the authors, without undue reservation, to any qualified researcher.

## Author Contributions

AM conceived and designed the experiments, collected, analyzed, and interpreted the data, and drafted the manuscript. MT conceived and designed the protocol 1 and critically revised the manuscript. SS collected, analyzed, and interpreted the data from protocol 1, and drafted the manuscript. GL collected, analyzed, and interpreted the data from protocol 2, and drafted the manuscript. WF and DM designed the experimental set-up, collected, analyzed, and interpreted the data from protocol 2. CF designed the protocol 2 and critically revised the manuscript. SR conceived and designed the experiments, and critically revised the manuscript. All authors approved the final version of the manuscript. The research was conducted at the Integrative Physiology Lab, Department of Neuroscience, University of Turin.

## Conflict of Interest Statement

The authors declare that the research was conducted in the absence of any commercial or financial relationships that could be construed as a potential conflict of interest.
